# Social position and economic system justification in Canada: Implications for advancing health equity and social justice from an exploratory study of factors shaping economic system justification

**DOI:** 10.3389/fpubh.2022.902374

**Published:** 2022-10-20

**Authors:** Kiffer G. Card, Kirk Hepburn

**Affiliations:** Faculty of Health Sciences, Simon Fraser University, Burnaby, BC, Canada

**Keywords:** status quo bias, economic system justification, health equity, wellbeing, social position

## Abstract

**Objective:**

Many socio-economic reforms that could reduce health disparities are not implemented because people justify existing systems and fear changes thereto. This study aimed to identify socio-demographic factors associated with system justifying beliefs to better understand how they are maintained in Canada. In doing so, we hypothesized that (1) systems justification is a default cognitive position, buttressed by the palliative benefits of system-justification, (2) lack of success in a given system generally motivates people to doubt the legitimacy of that system, and (3) system-justifying beliefs are rejected only when the costs of doing so are low enough and/or the benefits are high enough to outweigh the innate needs-fulfillment benefits of system-justification.

**Methods:**

Testing these hypotheses, we recruited participants living in Canada, aged 16+, to complete an online survey after being recruited *via* paid social media advertisements. Multivariable regression models identified factors associated with Economic System Justification Scale (ESJS) scores. Explanatory variables included demographic measures of social position, self-rated health, and patterns of social inclusion.

**Results:**

Among 2,619 participants, system-justifying beliefs were wide-spread, with the average level of support across ESJS scale items exceeding 50%. Lower ESJS scores were associated with worse health, more loneliness, and lower socioeconomic status. Despite the pattern that marginalization erodes system-justification, several historically marginalized characteristics (e.g., non-white ethnicity and non-binary gender) were associated with relatively high system-justification, compared to matching privileged characteristics (e.g., white ethnicity; masculine gender).

**Conclusion:**

Supporting our hypotheses, we identify a general trend that social marginalization is associated with less system-justification. Those benefitting from the status quo (e.g., healthier, wealthier, less lonely) were more likely to hold system-justifying beliefs. However, some groups who are disadvantaged within the existing system reported higher system-justification—suggesting that system oppression may be a key moderator of the effect of social position on system justification.

## Introduction

Many social and economic reforms that are designed to reduce health disparities are not implemented because they lack public support (1, 2). A common barrier to amassing public support for such policies is the public's preference for existing systems and aversion to change. This phenomenon has been referred to as “status quo bias” (3, 4). As an example, neoliberal beliefs about personal agency, behavior, responsibility, and accountability justify existing health and social systems because they presuppose that health disparities are the product of individual choices and not systemic inequalities; and therefore, changes to the system are not needed (5, 6).

According to System Justification Theory, biases in favor of the status quo—and the political ideologies that rationalize these biases—arise from basic human needs: the need for a general sense of stability, certainty, and predictability; the need to belong; the need to understand the world and one's place in it; and the need to feel good about one's self and community (7–9). Material and tangible benefits of social conformity and performativity also likely support system-justification. In other words, people are cognitively motivated to construct and uphold system-justifying beliefs because these beliefs aid and pacify innate human needs (10). Thus, ideological support for systems can be interpreted as a default *post-hoc* rationalization that allows individuals to benefit from existing systems and structures without cognitive dissonance for the harms these systems cause (11, 12). Unfortunately, this phenomenon poses a considerable obstacle to the sort of social and economic change that is needed to address health disparities (13).

Of course, there are many people who do not believe that the status quo is justified. So, how do we explain the emergence of these system-challenging beliefs in the presence of status quo bias? One explanation, informed by rational choice models of political behavior, is that people who are better pacified by a given system are more inclined to maintain system-justifying beliefs; while those who are disadvantaged by a given system are inclined to shed these beliefs and seek out reforms (14, 15). For example, while majorities of people widely believe health inequities are driven by traditional health determinants (e.g., *personal knowledge and health behaviors*), those from marginalized backgrounds are relatively more likely to endorse the importance of social determinants of health [e.g., *one's economic and social position* (16)]. This explanation supports the basic premise of System Justification Theory—that political ideology represents a form of motivated social cognition (17, 18)—while also helping us to understand the correlation of social position and system-justification. Indeed, while people generally accept system justifying beliefs (*such as the belief that health is driven by individual and not systemic causes*), their lived experience appears to override status quo bias. This may be because the cognitive and personal costs of system-justifying ideologies outweigh the benefits of supporting the system when one's position in it is disadvantaged (19).

While it is plausible that social position may provide a cognitive motive for rejecting system justification, several studies have found that systematically oppressed individuals in a given system are actually more likely to hold system-justifying beliefs compared to those with relative privilege (20–22). For example, data from Pew Research Center shows that Black Democrats are considerably more moderate than white Democrats (23) and the Survey Center on American life shows that White liberals favor defunding the police more than Black and Hispanic Americans (24). Similarly, low-income uneducated white voters are more likely to support Republicans than high-income educated white voters (25). Finally, van der Toorn et al. (9) showed that people who feel most powerless believe most strongly in the legitimacy of governments (26). In each of these comparisons, social marginalization appears to be associated with stronger system-justifying beliefs: giving rise to what has been referred to as the “status legitimacy hypothesis” (22)—which is a surprising contradiction to our rationale choice hypothesis that people underserved by a system will be motivated to hold beliefs that support system-change.

Based on these various (seemingly contradictory) findings, three hypotheses are advanced about system-justifying beliefs: First, status quo bias is a default cognitive position, buttressed by the palliative benefits of system-justification (8). Second, lack of success in a given system generally motivates people to doubt the legitimacy of that system (27). Third, system-justifying beliefs are rejected only when the costs of doing so are low enough and/or the benefits are high enough to outweigh the innate needs-fulfillment benefits of system-justification. This three-part hypothesis may explain why some markers of social disadvantage are associated with high levels of system justification, despite the general trend that people underserved by a system are motivated to doubt its legitimacy. Put another way, marginalization promotes system- challenging beliefs, until it doesn't—until it oppresses these beliefs. For example, if challenging the system comes at a higher price for marginalized individuals than it does for privileged individuals, our three-part hypothesis would suggest that the marginalized group would be more strongly motivated to justify the system. In other words, the privilege of belonging to a privileged group would allow one, ironically, the freedom to reject the system which privileges her. Conversely, oppressed minorities may be oppressed into acceptance of the status quo. If true, conflicting findings about the status-legitimacy hypothesis likely arise from specific social processes within specific systems (22). It is thus important to identify which markers of social position are associated with system-justifying and system-challenging beliefs to understand the underlying social processes that must be addressed to generate consensus about the need for social change. Therefore, the present study aims to explore which dimensions of social position facilitate the rejection of system-justifying beliefs and which are associated with higher system-justification. In so doing, this exploratory study will (1) add to the empirical evidence regarding System Justification Theory, (2) empirically explore the validity of the status-legitimacy hypothesis (which posits higher system-justification among marginalized individuals compared to privileged individuals), and (3) demonstrate the relevance of these theories to the contemporary Canadian context.

## Methods

### Study setting

#### Study context

The present study aims to explore system justifying beliefs in Canada. While Canada is a relatively free, liberal democracy, it is also strongly influenced by the white, Anglo-Saxon, Protestant heritage of the settler-colonial government (28, 29). Canada's social and political system shares much with its southern neighbor, the United States, though Canada's political history and current trajectory has created a different system of social relations—particularly in the development of its conservative political movement (30). For example, religious, anti-state conservativism in Canada is significantly less potent (30–32)—leading to radically different outcomes across several leading cultural contests [e.g., gay marriage, gun control, abortion, unions, immigration (33–36)]. Further, all of the country's major political parties espouse support for pluralism, multiculturalism, and social equality (37–40); and since the 1980s, the *Canadian Charter of Rights and Freedoms* has provided a robust modern framework for human rights protection in Canada (41). Nevertheless, it is well-documented that the Canadian system favors mainstream, populist interests (42, 43) that marginalize Black, Indigenous, Muslim, French-speaking, and other racialized minorities and ethnic groups (40, 44, 45). Despite this, Canada's centrist party (i.e., the Liberal Party) continues to out-compete relatively more progressive entities for electoral support from these groups—a fact that boosts its reputation as “Canada's natural governing party” (46–48). Notably, social mobility is declining in Canada and inequality is increasing—though Canada compares relatively well on both indicators to the United States (49, 50). In a June 2020 report from the Parliamentary Budget Officer, the top 1% of Canadians control approximately one-fourth of the nation's wealth (51)—and the politics of wealth distribution in the country have remained relatively stagnant since the 1990s (52). Given these realities, Canada provides a unique and interesting setting to study system-justification and test the hypotheses outlined in this paper's introduction.

### Data collection

Data for this study was drawn from an online convenience sample conducted between May and June 2020. Participants were residents of Canada, 16 years of age or older, who were recruited using paid advertisements on Facebook and Instagram. Participants who completed the survey were entered into a prize drawing for $400 CAD. Advertisements were posted in English and French and directed participants to a Qualtrics survey available in either language. Upon initiating the survey, each participant was screened for eligibility, provided informed consent, and completed a 20-min questionnaire.

#### Outcome variable

**Figure 1:**
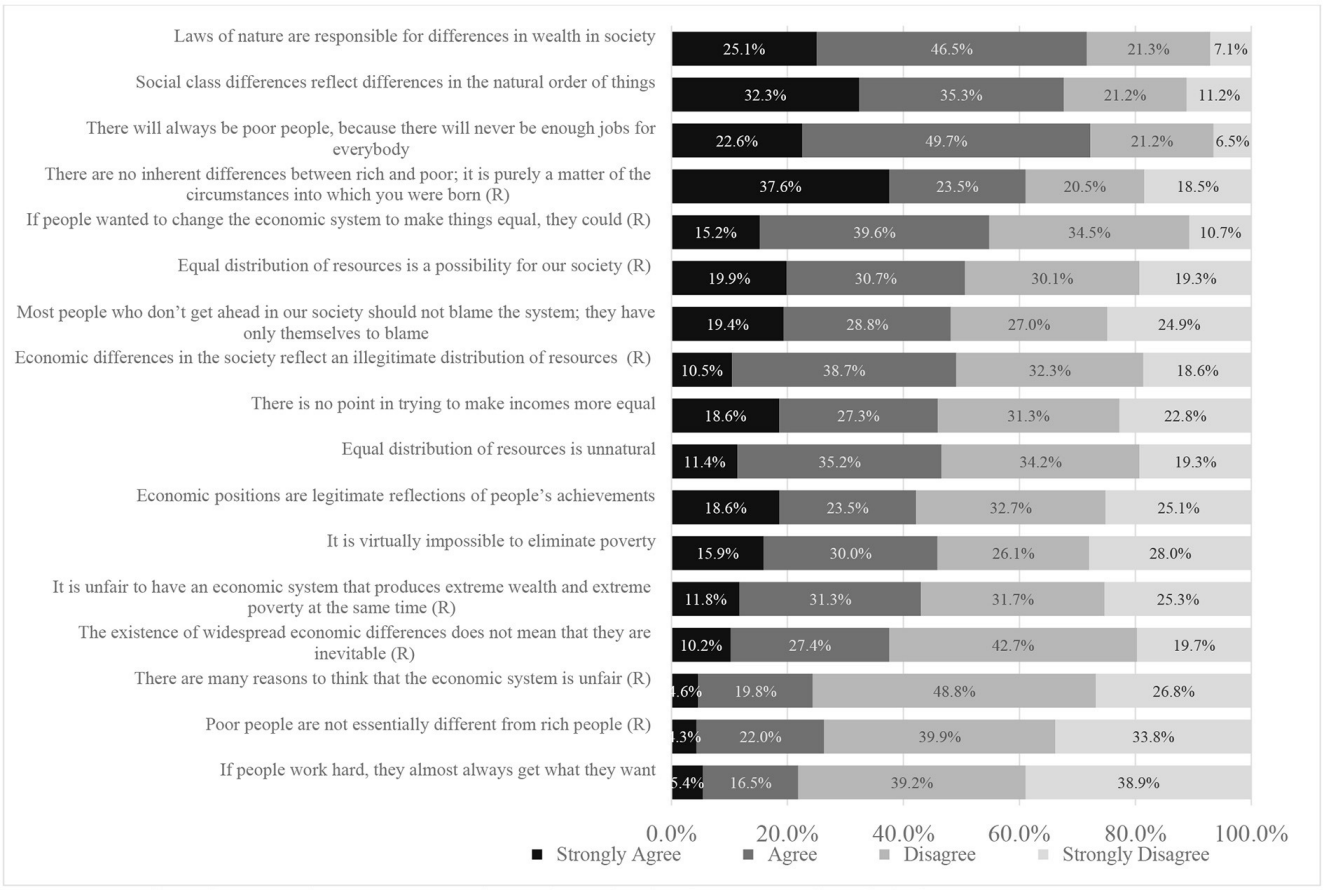
Economic system justification scale (ESJS) items. (R), Reverse Scored (i.e., Higher agreement indicates lower levels of system-justifying belief).

The Economic System Justification Scale (ESJS) was used as a general measure of system-justifying beliefs (53). Scale items for the ESJS are provided in Figure 1 and were scored on a 4-point Likert scale: Strongly Agree, Agree, Disagree, Strongly Disagree. In brief, these items measured participant's attitudes about the legitimacy (e.g., “*Economic differences in the society reflect an illegitimate distribution of resources*”), naturality (e.g., “*Laws of nature are responsible for differences in wealth in society*”), and inevitability (e.g., “*It is virtually impossible to eliminate poverty*”) of the economic system. The Cronbach alpha score for the scale was found to be high (α = 0.90) and final scale scores range from 17 (lower system justification) to 68 (higher system-justification).

#### Explanatory variables

To measures aspects of a participant's social position, a demographic questionnaire was completed. This questionnaire assessed participant's age, gender identity, sexual orientation, ethnicity, relationship and family status, disability status, income level, education level, occupation classification, housing situation, province of residence, geographic rurality-urbanity, religious affiliation. These factors were selected from a list of pre-determined options, aligned with the Canadian Census. Participant's self-rated health and experiences of loneliness were also included. To assess self-rated health participants were asked “In general, would you say your health is…Excellent, Very Good, Good, Fair, or Poor?” The 3-item UCLA loneliness scale [Study α = 0.86; (54)] was used to assess loneliness. This scale asks participants how often they feel they “lack companionship?” “left out?” and “isolated from others?” Each question is scored on a three-point scale (1) “Hardly Ever,” (2) “Some of the time,” or (3) “Often.” The sum of scale items is calculated, and higher scores represent greater loneliness.

All variables were self reported except the geographic rurality and urbanity, which was based on data from the Canadian Census and linked to participant responses using their forward sortation area code (i.e., the first three digits of their Canadian postal code). Based on World Bank practices, participants living in forward sortation areas with a population of <300 people per square kilometer were classified as being rural, participants living in regions with more than 1,500 residents per square kilometer were classified as being urban, and participants living in between these values were classified as being suburban (55).

### Data analysis

Analyses were conducted in R version 4.1.2. (56). Multiple imputation using fully conditional specification implemented using the MICE algorithm described by Buuren and Groothuis-Oudshoorn (57) was used to impute missing data on explanatory variables using the mice package (57, 58). This approach allowed for imputation of each variable independently using regression based equations. For all variables, five imputations were conducted using the cart method (i.e., classification and regression trees, with up to 250 iterations per imputation. Imputation did not affect the overall findings in sensitivity analyses—suggesting that data are missing mostly at random. Observations missing the primary outcome variable were removed using listwise deletion.

To address our three hypotheses we sought to (a) examine the prevalence of system justifying beliefs by examining the data descriptively to identify how widespread support was for economic system justifications and (b) identify the characteristics that were associated with greater system justification using a multivariable framework that allowed us to identify the independent effects of each sociodemographic factor. These multivariable results were then interpreted qualitatively in order to see if greater success within the system was associated with higher system justification and assess whether any marginalized populations were more likely to endorse the system compared to their relatively privileged counterparts. Descriptive statistics were calculated for the overall sample using the tableone package (59) and the psych package (60). Separate bivariable regression models tested associations between each explanatory variable and ESJS scores. Based on these results two multivariable linear regression were conducted using base R's glm function. A linear regression was selected due to normality of distributed outcome variable and confirmation of linear regression assumptions being met in standard diagnostic plots. For multivariable regression analyses, ESJS scores were treated as the continuous outcome variable and all other variables were treated as explanatory factors. This approach enabled us to identify the independent and adjusted factors associated with system-justifying beliefs. As mentioned above, two multivariable models were constructed: the first included all variables of theoretical interest, and the second was built using variables selected *via* stepwise backwards selection for AIC minimization. AIC minimization was used to balance model simplicity and explanatory power. Results from the stepwise selected model are discussed. Notably, the full model and stepwise selected model had similar *R*^2^-values (0.313 vs. 0.312), the differences in AIC were small (18,622 vs. 18,620), and the general conclusions reached from the models did not appear to be sensitive to the model building approach. Based on regression results, boxplots were created using the ggplot package to illustrate important relationships between key variables of interest (61).

Several additional *post-hoc* analyses were conducted by constructing boxplots to examine relationships between ESJS scores and key variables. This was done to better understand the results of the multivariable models and provide further insights into possible inter-relationships between variables, consistent with an intersectional analysis approach (62). The first, examines ESJS scores across political party affiliations. The second, examined levels of ESJS scores by health status and income. The Third, examined ESJS scores by ethnicity and educational attainment.

## Results

Descriptive statistics for the analytic sample are provided in Table 1. In summary, among 2,619 eligible participants, the median age of our sample was 60.2 years. The sample was disproportionately composed of people who were women (53.6%), identified as white (74.8%), were straight/heterosexual (83.5%), were in a relationship (67.7%), had a college education (41.8%), and had incomes of $60,000 or more (53.0%). Most participants also reported owning their home (67.7%) and having good health (72.7%). Approximately one-third of participants reported living with an auditory, visual, physical, cognitive, or other disability (37.1%), half (48.6%) identified as living in rural regions of Canada, and half reported being Christian (50.9%). With respect to our first hypothesis (i.e., that system justification beliefs are widespread), we found system justifying beliefs were widespread with the average level of support across all items being 53.8% and a median score of 39 (representing a slight tendency for agreement within the whole sample).

**Table 1:** Descriptive statistics.

	**Overall**	**ESJS score > 39**	**ESJS score <39**
	**(*N* = 2,619)**	**(*N* = 1,302)**	**(*N* = 1,317)**
**Age** ***Mean (SD)***	60.2 (13.7)	59.9 (14.1)	60.5 (13.3)
<30	100 (3.8)	64 (4.9)	36 (2.8)
31–59	902 (34.6)	427 (32.9)	475 (36.2)
60+	1,608 (61.6)	808 (62.2)	800 (61.0)
**Gender**			
Man	1,024 (39.1)	336 (25.8)	688 (52.2)
Non-Binary	190 (7.3)	53 (4.1)	137 (10.4)
Woman	1,405 (53.6)	913 (70.1)	492 (37.4)
**Ethnicity**			
African, Caribbean, or Black	67 (2.6)	21 (1.6)	46 (3.5)
East Asian	32 (1.2)	12 (0.9)	20 (1.5)
Indigenous	140 (5.3)	60 (4.6)	80 (6.1)
Other	391 (14.9)	117 (9.0)	274 (20.8)
South Asian	19 (0.7)	11 (0.8)	8 (0.6)
West Asian	11 (0.4)	3 (0.2)	8 (0.6)
White	1,959 (74.8)	1,078 (82.8)	881 (66.9)
**Sexual orientation**			
Heterosexual	2,187 (83.5)	1,105 (84.9)	1,082 (82.2)
Gay, Lesbian, Bisexual, Queer, or Other	432 (16.5)	197 (15.1)	235 (17.8)
**Relationship status**			
Single	846 (32.3)	511 (39.2)	335 (25.4)
In a relationship	1,773 (67.7)	791 (60.8)	982 (74.6)
**Educational attainment**			
High school diploma or lower	425 (16.2)	203 (15.6)	222 (16.9)
Advanced training below bachelor level	1,100 (42.0)	539 (41.4)	561 (42.6)
Bachelors or higher	1,094 (41.8)	560 (43.0)	534 (40.5)
**Occupation & employment status**			
Management, finance, and administration	288 (11.0)	112 (8.6)	176 (13.4)
Arts, culture, and sport	46 (1.8)	25 (1.9)	21 (1.6)
Education, law, and government	249 (9.5)	141 (10.8)	108 (8.2)
Health and science	305 (11.6)	174 (13.4)	131 (9.9)
Manufacturing, trades, and resource	257 (9.8)	70 (5.4)	187 (14.2)
Sales and services	200 (7.6)	99 (7.6)	101 (7.7)
Retired	1,164 (44.4)	600 (46.1)	564 (42.8)
Student	37 (1.4)	26 (2.0)	11 (0.8)
Unemployment/disability	51 (1.9)	43 (3.3)	8 (0.6)
Unpaid care giving	22 (0.8)	12 (0.9)	10 (0.8)
**Household income**			
<$29,999	537 (20.5)	342 (26.3)	195 (14.8)
$30,000–$59,999	693 (26.5)	367 (28.2)	326 (24.8)
$60,000–$89,999	547 (20.9)	255 (19.6)	292 (22.2)
$90,000 or more	842 (32.1)	338 (26.0)	504 (38.3)
**Housing situation**			
Own	1,774 (67.7)	781 (60.0)	993 (75.4)
Rent	691 (26.4)	430 (33.0)	261 (19.8)
Other	154 (5.9)	91 (7.0)	63 (4.8)
UCLA loneliness score *Mean (SD)*	4.97 (1.95)	5.45 (2.01)	4.50 (1.77)
**Self-rated health**			
Poor	219 (8.4)	153 (11.8)	66 (5.0)
Fair	497 (19.0)	301 (23.1)	196 (14.9)
Good	930 (35.5)	458 (35.2)	472 (35.8)
Very good	735 (28.1)	320 (24.6)	415 (31.5)
Excellent	238 (9.1)	70 (5.4)	168 (12.8)
**Disability status**			
No	1,648 (62.9)	738 (56.7)	910 (69.1)
Yes	971 (37.1)	564 (43.3)	407 (30.9)
**Religious affiliation**			
Protestant	615 (23.5)	262 (20.1)	353 (26.8)
Catholic/Orthodox	424 (16.2)	172 (13.2)	252 (19.1)
Other Christian	294 (11.2)	89 (6.8)	205 (15.6)
Non-Christian	346 (13.2)	209 (16.1)	137 (10.4)
Agnostic	626 (23.9)	359 (27.6)	267 (20.3)
Atheist	314 (12.0)	211 (16.2)	103 (7.8)
**Rurality-Urbanity**			
Urban	796 (30.4)	412 (31.6)	384 (29.2)
Rural	1,272 (48.6)	616 (47.3)	656 (49.8)
Suburban	551 (21.0)	274 (21.0)	277 (21.0)
**Region**			
Ontario	876 (33.4)	467 (35.9)	409 (31.1)
Atlantic Canada	244 (9.3)	142 (10.9)	102 (7.7)
British Columbia	599 (22.9)	298 (22.9)	301 (22.9)
Prairies	758 (28.9)	304 (23.3)	454 (34.5)
Quebec	129 (4.9)	87 (6.7)	42 (3.2)
Territories	13 (0.5)	4 (0.3)	9 (0.7)

Table 2 provides bivariable and multivariable results identifying the independent and adjusted factors associated with higher levels of economic system-justifying beliefs (as measured using ESJS scores). Regression coefficients and 95% confidence intervals are reported in table format, but not repeated in-text. With respect to our second hypothesis (e.g., that success within a system promotes system justification), our multivariable results showed that higher ESJS scores were associated with lower levels of loneliness and higher self-rated physical health. With respect to our third hypothesis (e.g., that some marginalized groups would be deterred from system-rejection due to high social costs), identifying as gender non-binary (vs. identifying as a man), non-white ethnic identification [i.e., African, Caribbean, or Black; Arab/West Asian; Indigenous; or Other Ethnic Orientation (vs. White)], higher income, better self-rated health, reporting a Christian religious affiliation, or living in the Prairie region of Canada. Lower ESJS scores were associated with identifying as a woman (vs. man); having a Bachelor's degree or higher level of education, working in civic services (i.e., Education, Law, Government, Health and Science); being retired; being a student; renting (vs. owning); reporting a non-Christian religious affiliation (i.e., Atheist, Agnostic, or Other Non-Christian Religious Tradition); and residence in Quebec.

**Table 2:** Regression models identifying associations with higher economic social justification scale scores.

	**Bivariable models testing associations between each explanatory variable and ESJS scores**		**Multivariable models testing associations between all backwards stepwise selected variables and ESJS scores**	
	**β**	**95% CI**	**β**	**95% CI**
**Age**	0.02	−0.01, 0.05	Not selected	
**Gender**				
Man	Reference		Reference	
Non-Binary	1.32	−0.13, 2.76	**1.68**	**0.01, 3.36**
Woman	**−7.13**	**−7.88**, **−6.37**	**−5.4**	**−6.12**, **−4.68**
**Ethnicity**				
White	Reference		Reference	
African, Caribbean, or Black	**6.95**	**4.57, 9.33**	**7.00**	**4.87, 9.13**
Arab/West Asian	**6.98**	**1.19, 12.77**	**7.02**	**1.97, 12.07**
East Asian	3.07	−0.34, 6.49	1.87	−1.12, 4.85
Indigenous	**3.18**	**1.51, 4.86**	**2.83**	**1.36, 4.30**
South Asian	0.65	−3.77, 5.07	2.22	−1.62, 6.05
Other	**5.8**	**4.74, 6.86**	**3.42**	**2.44, 4.41**
**Sexual orientation**				
Heterosexual	Reference		Reference	
Gay, Lesbian, Bisexual, Queer, or Other	**1.04**	**0.01, 2.08**	−1.22	−2.38, −0.06
**Relationship status**			Not selected	
Single	Reference			
In a relationship	**3.43**	**2.62, 4.24**		
**Educational attainment**				
High school diploma or lower	Reference		Reference	
Advanced training below bachelor level	0.53	−0.59, 1.66	−0.33	−1.29, 0.64
Bachelors or above	−0.24	−1.36, 0.89	**−2.08**	**−3.11**, **−1.06**
**Occupation & employment status**				
Management, finance, and administration	Reference		Reference	
Arts, culture, and sport	**−3.37**	**−6.42**, **−0.32**	0.06	−2.60, 2.73
Education, law, and government	**−4.76**	**−6.42**, **−3.1**	**−2.84**	**−4.28**, **−1.40**
Health and science	**−4.11**	**−5.69**, **−2.53**	**−1.98**	**−3.34**, **−0.61**
Manufacturing, trades, and resource	**2.43**	**0.78, 4.08**	0.72	−0.73, 2.17
Sales and services	**−2.45**	**−4.22**, **−0.69**	−0.49	−2.04, 1.06
Retired	**−2.78**	**−4.04**, **−1.51**	**−1.26**	**−2.40**, **−0.12**
Student	**−7.37**	**−10.73**, **−4.01**	**−3.94**	**−6.89**, **−1.00**
Unemployment/Disability	**−8.79**	**−11.71**, **−5.87**	−2.46	−5.06, 0.14
Unpaid care giving	−3.65	−7.9, 0.6	0.58	−3.10, 4.26
**Household income**				
<$29,999	Reference		Reference	
$30,000–$59,999	**2.42**	**1.31, 3.53**	0.8	−0.19, 1.79
$60,000–$89,999	**3.59**	**2.41, 4.76**	**1.59**	**0.50, 2.68**
$90,000 or more	**5.02**	**3.96, 6.09**	**1.44**	**0.36, 2.52**
**Housing situation**				
Own	Reference		Reference	
Rent	**−3.64**	**−4.51**, **−2.77**	**−1.25**	**−2.09**, **−0.41**
Other	**−3.3**	**−4.94**, **−1.67**	−1.2	−2.65, 0.25
**UCLA loneliness score**	**−1.33**	**−1.52**, **−1.14**	**−0.59**	**−0.78**, **−0.41**
**Self–rated health**				
Poor	Reference		Reference	
Fair	**2.09**	**0.53, 3.64**	0.48	−0.91, 1.87
Good	**3.82**	**2.38, 5.26**	0.67	−0.68, 2.02
Very good	**5.54**	**4.06, 7.02**	**1.8**	**0.39, 3.21**
Excellent	**9.42**	**7.63, 11.22**	**3.58**	**1.87, 5.29**
**Disability Status**				
No	Reference		Reference	
Yes	**−2.83**	**−3.62**, **−2.04**	−1.13	−1.84, −0.43
**Religious affiliation**				
Protestant	Reference		Reference	
Catholic/Orthodox	0.82	−0.38, 2.02	0.16	−0.90, 1.21
Non-Christian	**−4.1**	**−5.38**, **−2.82**	**−4.12**	**−5.28**, **−2.96**
Agnostic	**−2.55**	**−3.63**, **−1.47**	**−2.51**	**−3.46**, **−1.56**
Atheist	**−5.21**	**−6.52**, **−3.89**	**−4.84**	**−6.00**, **−3.68**
Other Christian	**3.42**	**2.07, 4.77**	**1.71**	**0.52, 2.91**
**Rurality-Urbanity**			Not selected	
Urban	Reference			
Rural	0.7	−0.19, 1.58	
Suburban	0.63	−0.46, 1.72	
**Region**				
Ontario	Reference		Reference	
Atlantic Canada	−0.6	−2.01, 0.81	0.17	−1.04, 1.37
British Columbia	**1.14**	**0.11, 2.18**	0.83	−0.06, 1.71
Prairies	**2.98**	**2.01, 3.94**	**1.58**	**0.75, 2.41**
Quebec	**−3.25**	**−5.08**, **−1.41**	**−2.62**	**−4.19**, **−1.05**
Territories	4.72	−0.71, 10.15	2.26	−2.39, 6.91

Bold values indicate *p* < 0.05.

**Figure 2:**
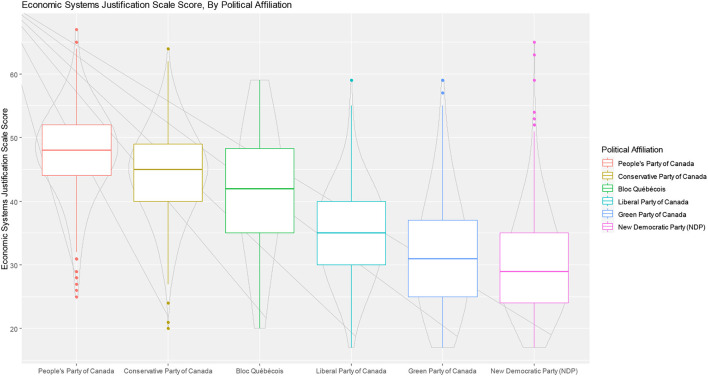
Boxplots of ESJS scores, by political party affiliation.

**Figure 3:**
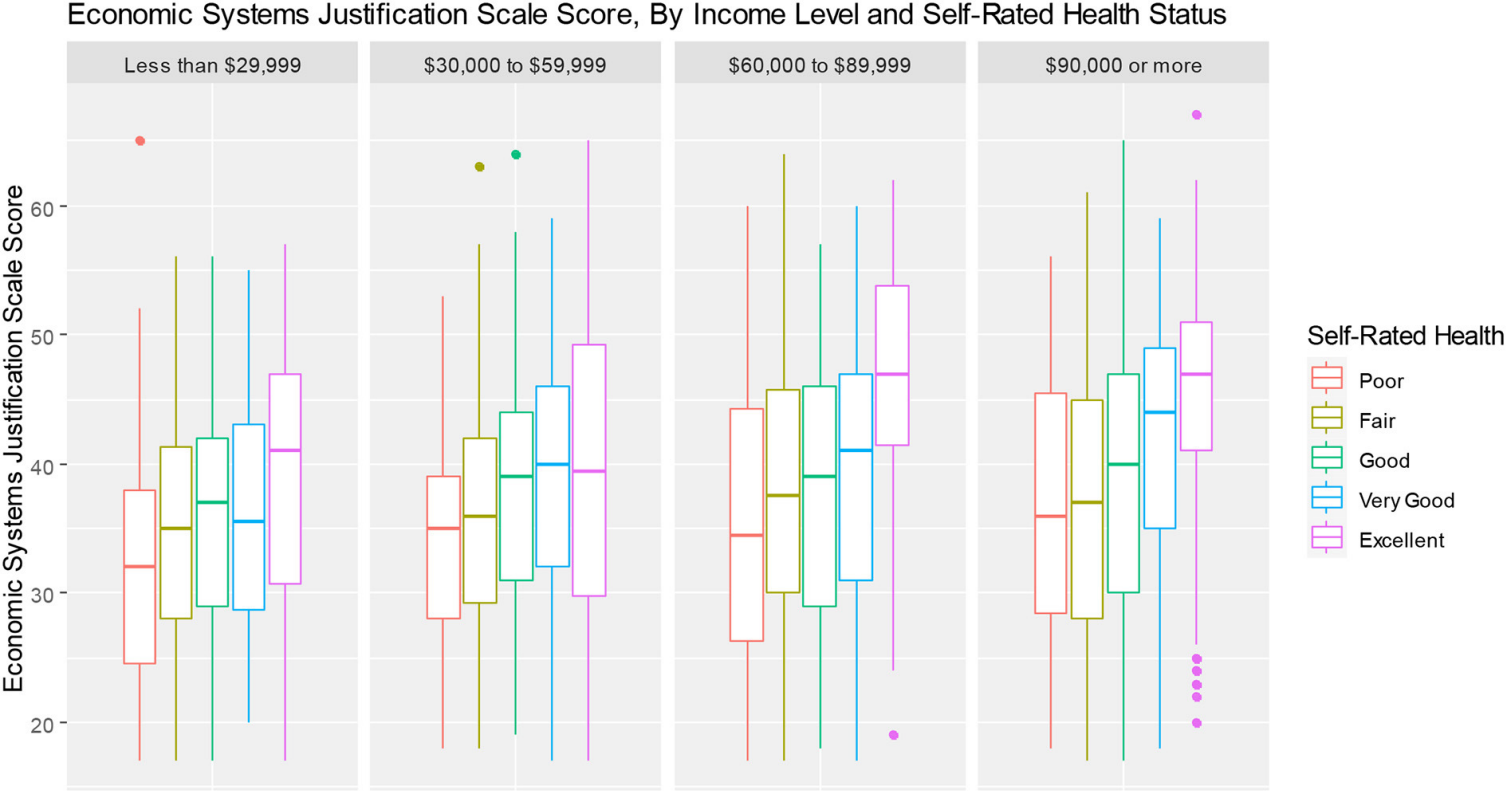
System justification scale scores, by income level and self-rated health status.

**Figure 4:**
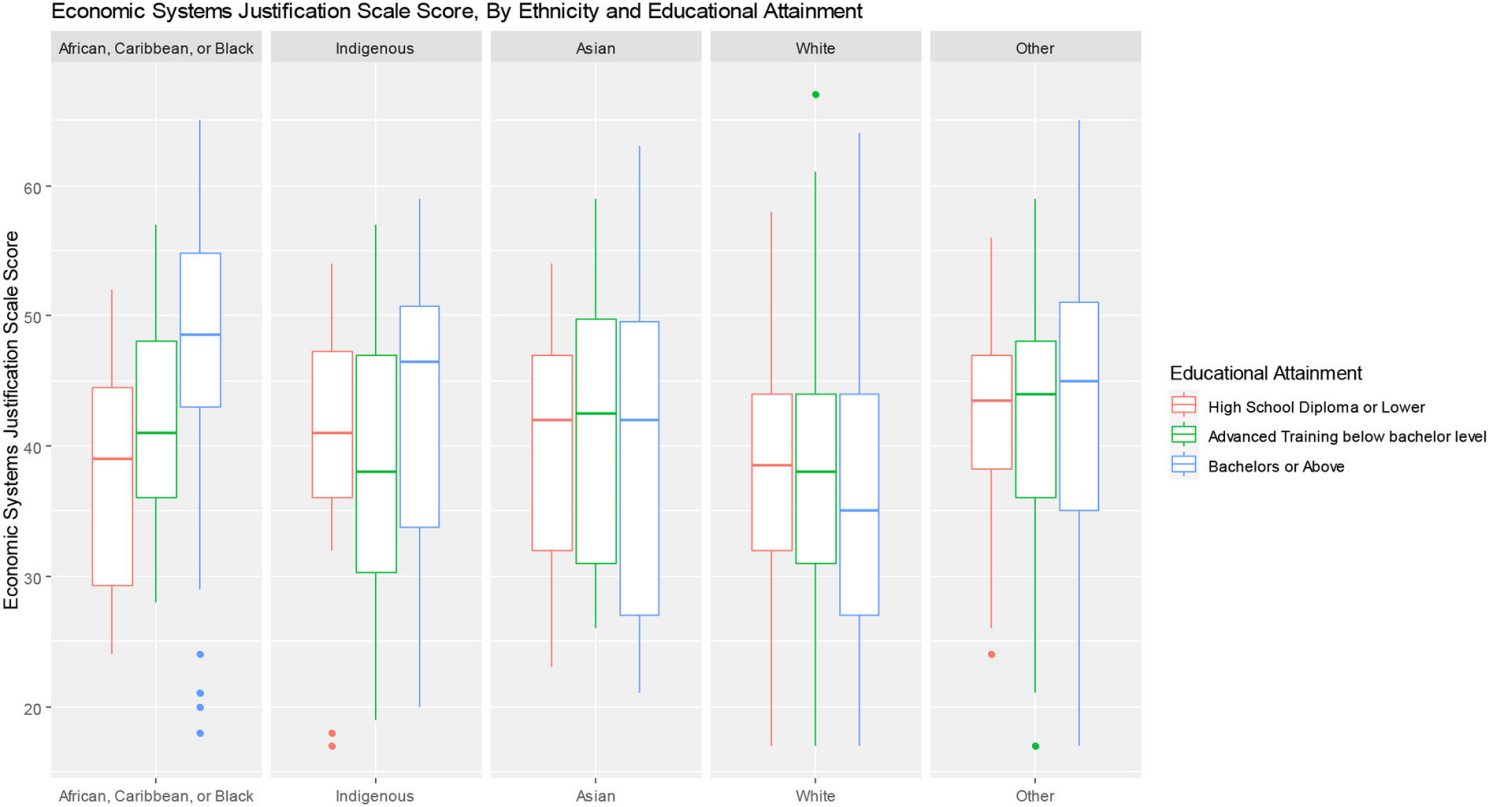
System justification scale scores, by ethnicity and educational attainment.

*Post-hoc* analyses of ESJS scores interesections with key variables are presented as boxplots in Figures 2, 3. Figure 2 shows that endorsement of ESJS scores are correlated with political party affiliation, but with considerable overlap of data. Figure 3 shows that within each income group, higher self-rated physical health is associated with higher economic system justification. Figure 4 shows that the relationship between educational attainment and ESJS scores differ by ethnicity—particularly for African, Caribbean, and black people for whom there appears to be a positive relationship between educational attainment and higher ESJS scores.

## Discussion

### Primary findings and relationship to existing studies

The present study aimed to examine which dimensions of social position were associated with system-justifying beliefs. Our intention was to understand better how system justification arises and prevents the emergence of reforms that would promote health equity for marginalized people and the overall improvement of health among Canadians. In doing so, three hypotheses were advanced about system justifying beliefs suggesting (1) that system-justification beliefs would be widespread—reflecting their role as a default bias in favor of the status quo; (2) that system-justifying beliefs would be lower among people whose needs were less well-met under the status quo, and (3) that some marginalized groups would nevertheless hold stronger system-justifying beliefs compared to privileged groups due to marginalizing processes that increase the costs and/or reduce the benefits of challenging the system. Results from our study generally supported these hypotheses, as discussed below:

### Hypothesis 1: System justifying beliefs are widespread

Examining the first hypothesis, we find that system-justifying beliefs are widespread. For example, when looking at the item response patterns for the ESJS, 8 of the 17 scale items had at least half of respondents support the system-justifying position. Given that Canada's political system is a multi-party parliamentary system, this level of support for these items is significant and cuts through political party divides (See Figure 2 for boxplots showing ESJS scores, stratified by political party affiliation). The wide-spread nature of system-justifying beliefs supports the assertion of systems justification theory that people from across all strata and segments of society actively participate in the upholding of established social and economic relations in Canada (11). This finding has major implications for understanding how the bias toward the status quo undermines the advancement of equity-oriented policies that would improve the health and wellbeing of Canadians.

### Hypothesis 2: Those benefiting from a system show more support for the system

That said, results regarding our second hypotheses indicated that participants who were less well off in the Canadian system generally had lower ESJS scores—meaning they were less likely to hold system justifying beliefs. Indeed, poorer health, higher loneliness, lower income, and renting instead of owning one's home were all associated with lower propensity to hold system-justifying beliefs. These effects appear to be compounding—as shown in Figure 3, which shows increasing levels of system justification, by health status, within income groups. These findings support System Justification Theory, which predicts people would be benefited by justifying the system (11). Even though the causal pathways are not easily identified in our cross-sectional data, our study does indicate that the relationship between system justification and wellbeing are wide-ranging—affecting multiple life domains. A circular, feedback-loop style of causation is likely implied (i.e., people who hold system justifying beliefs thrive in the system, and this thriving reinforces their system-justifying behavior)—though future longitudinal cross-lagged panel models would be helpful in establishing the presence of this causal pattern (63).

However, it is important to note the instances in which indicators of “success” were associated with less systems-justifying belief. These included higher educational attainment and being a student—factors which may highlight opportunities for the continued and expanded use of public education as a means of overcoming status quo bias (64). However, as shown in Figure 4, we should not necessarily assume that the radicalizing force of education works the same for all identity groups. These data show that while higher education might be associated with lower system justification for white individuals, there appears to be a positive association for African, Caribbean, and Black individuals—perhaps because Black youth who do not behave in accordance with system-justifying beliefs are less likely to be admitted by educational institutions or perhaps those who are recognize and attribute their success to the tremendous efforts required to gain admission and success within the educational system. These associations may highlight the tendencies of some life experiences to either promote or discourage system-justification—and that the effects may be modified according to one's social position. Understanding differences in these dynamics between demographic groups may be critical for effective messaging that can help individuals to rationalize interventions that would promote health equity. In particular, these dynamics promote the need for community-based and culturally-aware interventions that seek to build community support within key populations.

Furthering underscoring the importance of understanding these nuanced dynamics, our findings regarding occupation showed lower ESJS scores among people working in education, law, health, science, and government, highlighting the ways that potential pressures within one's everyday social environment may inform the emergence of system-justifying or system-challenging beliefs. The role of occupation may be especially important for further research, given the prominent social and political role that some industries can play in shaping Canadian policy and the extent to which cultural deviation within occupational cultures could limit the success of dissenting individuals within these micro-cultures (65–67). Nevertheless, despite these situational factors—and even controlling for them in the multivariable model—our findings generally support the second tenet of our hypothesis, which predicted higher system-justification among more successful individuals and lower system-justification among those who were marginalized by mainstream expectations of health and success. Recognizing how these personal motives drive support or rejection for a system, educators and activists should adopt an empathetic and conversational approach that can help individuals understand how our different life experiences might inform our world views (68). Doing so may help people recognize the value of lived experience in understanding the need for creating systems which help a greater share of the population and therby promote health equity.

### Hypothesis 3: Systems rejection is suppressed in some groups for whom costs may be too high

Regarding our third hypothesis, results show that some marginalized groups had higher ESJS scores compared to their relatively less-marginalized comparators. For example, non-binary and non-white individuals (i.e., African, Caribbean, or Black; Arab/West Asian; Indigenous; Other ethnicity) had stronger system-justifying beliefs than men and white people, respectively. However, not all marginalized identity groups had elevated ESJS scores compared to their privileged counterparts. For example, non-Christians had lower ESJS scores than Christians, despite the plurality of Canadians reporting Christian affiliation. Similarly, women had lower ESJS scores compared to men—despite historical and present-day sexism against women. Finally, people living in Quebec also had lower ESJS scores compared to those living in Ontario—despite historical tensions between Quebec and the Federal government.

Each of these findings is worthy of further sociological examination and a number of hypotheses could be advanced to explain these observations. For example, the oppression of gender minority and non-white individuals very likely increases the costs of desisting from system-justifying beliefs as is the case when minority political candidates are judged as more extreme compared to white and male candidates (69)—increasing the social sanctions (costs) for holding “extreme” views. These pressures can give rise to politics of respectability—which are used to deflect social pressures targeting one's identity (70, 71). Indeed, historic legacies of Canadian colonialism, nationalism, patriarchy, racism, and paternalism have sought to create “docile bodies” that conform to and support the Canadian status quo (8, 72–74). If these socialization processes have been able to achieve their goals of oppressing their target groups, this provides one mechanism to understand the phenomenon of higher ESJS scores among historically marginalized groups.

The challenge then is to understand how some groups have successfully overcome these restraints? For example, lower ESJS scores among women—who have certainly been oppressed for thousands of years by patriarchal, man-dominated society—suggests that perhaps the success of the feminist movement may have provided a pattern for eroding system-justifying ideologies. If this is the case, it becomes hard to explain why similarly valiant civil rights efforts have failed to support system-challenging ideologies among racialized people? Perhaps the respectability politics inherent in these movements are a key moderator? (71, 75). Alternatively, the causal paths underlying our observations have less to do with the oppressed groups and more to do with the socializing processes of their comparators. For example, white people may have low ESJS scores because they are more privileged in the Canadian system to dissent, and men may have higher ESJS scores than women because the contemporary system has more effectively brought men under the control of contemporary systems of patriarchy and masculinity (76). Given the complexity of the relationships identified here, these separate phenomena must be examined further on a case-by-case basis. It is, of course, possible—or even likely—that social processes not considered here (e.g., social dominance, group identity) override the standard effects to create these unique cases. As such, further qualitative and quantitative research on system justifying beliefs is merited.

### Limitations

The present exploratory study has several limitations that can be addressed in follow-up research. This study relies on a convenience online sample and a cross-sectional survey design. Findings may therefore not be generalizable or representative. Our sample skews older than the Canadian population, which may be an artifact of the sampling procedures and self-selection—as well as our restriction to adults age 16 years or older. All findings thus require replication and collaboration through other studies and approaches (e.g., telephone surveys, in-person sampling). We also note that our questionnaire was limited in scope. A variety of other social processes could be explored as confounders, mediators, and moderators to explain the associations we analyzed. Analytically, our regression models have identified the independent and adjusted factors associated with system justification, but future analyses could adopt an intersectional approach (e.g., How does system justification relate to the monolithic cis-white-straight-male identity?) to better understand potential group dynamics that drive system-justification.

## Conclusion

Regardless of our study's limitations, our findings advance the literature on System Justification Theory and the status-legitimacy hypothesis and demonstrate the operationalization of gender, ethnicity, and other markers of social position in shaping system-justifying ideologies of people in Canada. Furthermore, we conclude with support for a proposed three-part hypothesis that advances the idea that system-justifying beliefs are widespread, that adverse life experiences degrade system justifying beliefs, and that this effect is moderated within key identity groups—perhaps by the force of oppression exerted upon these groups. While further evidence is needed, several lines of inquiry related to the role of social identity and historic oppression are opened to understand how and why some demographic and social identities lend themselves to support of the existing system and status quo. Advancing the cause of health equity will require changes to the Canadian system, and our research will hopefully help us to understand how to manage ideologies that are biased in favor of the status quo.

## Data availability statement

The raw data supporting the conclusions of this article will be made available by the authors, without undue reservation.

## Ethics statement

The studies involving human participants were reviewed and approved by Harmonized Research Ethics Board of British Columbia. Written informed consent from the participants' legal guardian/next of kin was not required to participate in this study in accordance with the national legislation and the institutional requirements.

## Author contributions

KC conceptualized the design of the study and undertook analyses. KH assisted with the interpretation of study results and writing of the final manuscript. All authors contributed to the article and approved the submitted version.

## Funding

This study was funded with support from the Canadian Foundation for AIDS Research. KC was supported with funding from a Michael Smith Scholar Award.

## Conflict of interest

The authors declare that the research was conducted in the absence of any commercial or financial relationships that could be construed as a potential conflict of interest.

## Publisher's note

All claims expressed in this article are solely those of the authors and do not necessarily represent those of their affiliated organizations, or those of the publisher, the editors and the reviewers. Any product that may be evaluated in this article, or claim that may be made by its manufacturer, is not guaranteed or endorsed by the publisher.

## References

[1] Burstein P. The impact of public opinion on public policy: a review and an Agenda. Polit Res Q. (2003) 56:29–40. 10.1177/106591290305600103

[2] Trein P Fuino M Wagner J. Public opinion on health care and public health. Prev Med Rep. (2021) 23:101460. 10.1016/j.pmedr.2021.101460 34285870 PMC8273192

[3] Fernandez R Rodrik D. Resistance to reform: status quo bias in the presence of individual- specific uncertainty. Am Econ Rev. (1991) 81:1146–55.

[4] Xiao Q Lam CS Piara M Feldman G. Revisiting status quo bias: replication of Samuelson and Zeckhauser (1988)?. Meta-Psychology. (2021) 5. 10.15626/MP.2020.2470

[5] Peeters R. Manufacturing responsibility: the governmentality of behavioural power in social policies. Soc Policy Soc. (2019) 18:51–65. 10.1017/S.147474641700046X

[6] Vera LF. The (mis)shaping of health: problematizing neoliberal discourses of individualism and responsibility. In:Hosseini SAH Goodman J Motta SC Gills BK, editors. The Routledge Handbook of Transformative Global Studies. Abington: Routledge (2020). 10.4324/9780429470325-16

[7] Jost JT Glaser J Kruglanski AW Sulloway FJ. Political conservatism as motivated social cognition. Psychol Bull. (2003) 129:339–75. 10.1037/0033-2909.129.3.339 12784934

[8] Jost JT. A quarter century of system justification theory: questions, answers, criticisms, societal applications. Br J Soc Psychol. (2019) 58:263–314. 10.1111/bjso.12297

[9] van der Toorn J Jost JT. Twenty years of system justification theory: introduction to the special issue on “Ideology and system justification processes”. Group Proc Intergroup Relat. (2014) 17:413–9. 10.1177/1368430214531509

[10] Vargas-Salfate S Paez D Khan SS Liu JH Gil de Zúñiga H. System justification enhances well-being: a longitudinal analysis of the palliative function of system justification in 18 countries. Br J Soc Psychol. (2018) 57:567–90. 10.1111/bjso.12254 29577342

[11] Jost JT. A Theory of System Justification. Cambridge, MA: Harvard University Press (2020). 10.4159/9780674247192

[12] Jost JT van der Toorn J. System justification theory. In: Handbook of Theories of Social Psychology. Vol. 2. Newbury Park, CA: Sage Publications Ltd. (2012). p. 313–43. 10.4135/9781446249222.n42

[13] Williams DR Costa MV Odunlami AO Mohammed SA. Moving upstream: how interventions that address the social determinants of health can improve health and reduce disparities. J Public Health Manage Pract. (2008) 14:S8–17. 10.1097/01.PHH.0000338382.36695.42 18843244 PMC3431152

[14] Bolsen T Palm R. Motivated Reasoning and Political Decision Making. Oxford (2019). 10.1093/acrefore/9780190228637.013.923

[15] Harsanyi JC. Rational-Choice models of political behavior vs. functionalist and conformist theories. World Polit. (1969) 21:513–38. 10.2307/2009665

[16] Towe VL May LW Huang W Martin LT Carman K Miller CE. Drivers of differential views of health equity in the U.S.: is the U.S. ready to make progress? Results from the 2018 national survey of health attitudes. BMC Public Health. (2021) 21:175. 10.1186/s12889-021-10179-z 33478438 PMC7817761

[17] Jost JT Amodio DM. Political ideology as motivated social cognition: behavioral and neuroscientific evidence. Motiv Emot. (2012) 36:55–64. 10.1007/s11031-011-9260-7

[18] Strupp-Levitsky M Noorbaloochi S Shipley A Jost JT. Moral “foundations” as the product of motivated social cognition: empathy and other psychological underpinnings of ideological divergence in “individualizing” and “binding” concerns. PLoS ONE. (2020) 15:e0241144. 10.1371/journal.pone.0241144 33170885 PMC7654778

[19] Yang SL Xu BX Yu F Guo YY. Revisiting the status-legitimacy hypothesis: concepts, boundary conditions, psychological mechanisms. J Pac Rim Psychol. (2019) 13. 10.1017/prp.2019.15

[20] Henry P Saul A. The development of system justification in the developing world. Soc Justice Res. (2006) 19:365–78. 10.1007/s11211-006-0012-x19505192

[21] Jost JT Pelham BW Sheldon O Ni Sullivan B. Social inequality and the reduction of ideological dissonance on behalf of the system: evidence of enhanced system justification among the disadvantaged. Euro J Soc Psychol. (2003) 33:13–36. 10.1002/ejsp.127

[22] Sengupta NK Osborne D Sibley CG. The status-legitimacy hypothesis revisited: ethnic-group differences in general and dimension-specific legitimacy. Br J Soc Psychol. (2015) 54:324–40. 10.1111/bjso.12080 25156504

[23] Gilberstadt H, Daniller, A,. Liberals Make up the Largest Share of Democratic Voters, but Their Growth has Slowed in Recent Years. Pew Research Center (2020). Available online at: https://www.pewresearch.org/fact-tank/2020/01/17/liberals-make-up-largest-share-of-democratic-voters/

[24] Cox D,. Crime, Policing, the Racial Divide on the Left. The Survey Center on American Life (2022). Available online at: https://www.americansurveycenter.org/crime-policing-and-the-racial-divide-on-the-left/

[25] Doherty C, Kiley, J, Tyson, A, Johnson, B,. The Parties on the Eve of the 2016 Election: Two Coalitions, Moving Further Apart. Pew Research Center—U.S. Politics & Policy (2016). Available online at: https://www.pewresearch.org/politics/2016/09/13/the-parties-on-the-eve-of-the-2016-election-two-coalitions-moving-further-apart/

[26] van der Toorn J Feinberg M Jost JT Kay AC Tyler TR Willer R. A sense of powerlessness fosters system justification: implications for the legitimation of authority, hierarchy, and government. Polit Psychol. (2015) 36:93–110. 10.1111/pops.12183

[27] Jost JT Becker J Osborne D Badaan V. Missing in (collective) action: ideology, system justification, and the motivational antecedents of two types of protest behavior. Curr Dir Psychol Sci. (2017) 26:99–108. 10.1177/0963721417690633

[28] Barker AJ. The contemporary reality of canadian imperialism: settler colonialism and the hybrid colonial state. Am Indian Q. (2009) 33:325–51. 10.1353/aiq.0.0054

[29] Kaufmann EP. The decline of the ‘WASP' in Canada and the United States. In:Kaufmann EP, editor. Routledge (2004). p. 61–83. Available online at: http://www.routledge.com/books/details/9780415315425/

[30] Hoover DR Martinez MD Reimer SH Wald KD. Evangelicalism meets the continental divide: mloral and economic conservatism in the United States and Canada. Polit Res Q. (2002) 55:351–74. 10.1177/106591290205500204

[31] Dilmaghani M. Canadian religious trends: secularization, polarization, or free-rider exclusion? Soc Compass. (2018) 65:626–49. 10.1177/0037768618800415

[32] Farney J. Social conservatives and party politics in Canada and the United States. In:Farney J, editor. Social Conservatives and Party Politics in Canada and the United States. Toronto, ON: University of Toronto Press (2017).

[33] Eidlin B. Class vs. special interest: labor, power, and politics in the United States and Canada in the twentieth century. Polit Soc. (2015) 43:181–211. 10.1177/0032329215571280

[34] Mauser GA Margolis M. The politics of gun control: comparing Canadian and American patterns. Environ Plan C Govern Policy. (1992) 10:189–209. 10.1068/c100189

[35] Muldoon M. The Abortion Debate in the United States and Canada: A Source Book. Abingdon: Routledge (2021). 10.4324/9781315860725

[36] Rayside D Wilcox C editors. Faith, Politics, and Sexual Diversity in Canada and the United States. Vancouver: UBC Press (2012).

[37] Berry JW Others A. Multiculturalism and Ethnic Attitudes in Canada. Ottawa, ON: Printing and Publishing, Supply and Services Canada (1977).

[38] Goodyear-Grant E Johnston R Kymlicka W Myles J. Federalism and the Welfare State in a Multicultural World. Montreal: McGill-Queen's Press (2019). 10.2307/j.ctvdtpjc7

[39] Hiebert D. Winning, losing, and still playing the game: the political economy of immigration in Canada. Tijdschrift Voor Econ Soc Geogr. (2006) 97:38–48. 10.1111/j.1467-9663.2006.00494.x

[40] Kwak LJ. “New Canadians are new conservatives”: race, incorporation and achieving electoral success in multicultural Canada. Ethnic Rac Stud. (2019) 42:1708–26. 10.1080/01419870.2018.1508734

[41] Abu–Laban Y Nieguth T. Reconsidering the constitution, minorities and politics in Canada. Can J Polit Sci. (2000) 33:465–97. 10.1017/S0008423900000160

[42] Mackey E. House of Difference: Cultural Politics and National Identity in Canada. Abingdon: Routledge (1998).

[43] Snow D Moffitt B. Straddling the divide: mainstream populism and conservatism in Howard's Australia and Harper's Canada. Commonwealth Comp Polit. (2012) 50:271–92. 10.1080/14662043.2012.692922

[44] Guimond S. Group socialization and prejudice: the social transmission of intergroup attitudes and beliefs. Euro J Soc Psychol. (2000) 30:335–54. 10.1002/(SICI)1099-0992(200005/06)30:3<335::AID-EJSP994>3.0.CO;2-V

[45] Zine J. Islam in the Hinterlands: Muslim Cultural Politics in Canada. Vancouver: UBC Press (2012).

[46] Falconer T,. Governing the “Government Party”: Liberal Party of Canada Leadership Conventions of 1948, 1958 1968 (2012). Available online at: https://uwspace.uwaterloo.ca/handle/10012/6924

[47] James P Kasoff MJ. Canadian Studies in the New Millennium. Toronto, ON: University of Toronto Press (2007)

[48] Kim J,. Beyond The Liberal Party: Immigrant Voting Behaviour in Canada. Undefined (2008). Available online at: https://www.semanticscholar.org/paper/BEYOND-THE-LIBERAL-PARTY%3A-IMMIGRANT-VOTING-IN-Kim/82e5947c899a76cb53840a6c077352532bc9eed5

[49] Corak M. Income inequality, equality of opportunity, intergenerational mobility. J Econ Perspect. (2013) 27:79–102. 10.1257/jep.27.3.79

[50] Government of Canada SC. Trends in Intergenerational Income Mobility and Income Inequality in Canada. Statistics Canada (2021). Available online at: https://www150.statcan.gc.ca/n1/pub/11f0019m/11f0019m2021001-eng.htm

[51] Wodrich N,. Estimating the Top Tail of the Family Wealth Distribution in Canada. Office of the Parliamentary Budget Officer (2020). Available online at: https://www.pbo-dpb.gc.ca/web/default/files/Documents/Reports/RP-2021-007-S/RP-2021-007-S_en.pdf

[52] Banting KG Myles J. Framing the new inequality: The politics of income redistribution in Canada. In:Banting K Myles J, editors. Income Inequality: The Canadian Story. Institute for Research on Public Policy (2016).

[53] Jost JT Thompson EP. Group-Based dominance and opposition to equality as independent predictors of self-esteem, ethnocentrism, and social policy attitudes among African Americans and European Americans. J Exp Soc Psychol. (2000) 36:209–32. 10.1006/jesp.1999.1403

[54] Hughes ME Waite LJ Hawkley LC Cacioppo JT. A short scale for measuring loneliness in large surveys. Res Aging. (2004) 26:655–72. 10.1177/0164027504268574 18504506 PMC2394670

[55] Dijkstra L,. How do We Define Cities, Towns, Rural Areas? World Bank (2020). Available online at: https://blogs.worldbank.org/sustainablecities/how-do-we-define-cities-towns-and-rural-areas

[56] R Core Team. R: A Language Environment for Statistical Computing. R Core Team (2021). Available online at: https://www.r-project.org/

[57] Buuren S Groothuis-Oudshoorn C. MICE: multivariate imputation by chained equations in R. J Stat Softw. (2011) 45:1–67. 10.18637/jss.v045.i03

[58] Buuren S van Groothuis-Oudshoorn K Vink G Schouten R Robitzsch A Rockenschaub P. mice: Multivariate Imputation by Chained Equations (3.14.0) (2021). Available online at: https://CRAN.R-project.org/package=mice

[59] Yoshida K, Bartel, A, Chipman, JJ, Bohn, J, McGowan, L, Da Barrett, M, . tableone: Create ‘Table 1' to Describe Baseline Characteristics With or Without Propensity Score Weights (0.13.0) (2021). Available online at: https://CRAN.R-project.org/package=tableone

[60] Revelle W. psych: Procedures for Psychological, Psychometric, Personality Research (2.1.9) (2021). Available online at: https://CRAN.R-project.org/package=psych

[61] Wickham H, Chang, W, Henry, L, Pedersen, TL, Takahashi, K, Wilke, C, . ggplot2: Create Elegant Data Visualisations Using the Grammar of Graphics (3.3.5) (2021). Available online at: https://CRAN.R-project.org/package=ggplot2

[62] Anthias F. Intersectional what? Social divisions, intersectionality and levels of analysis. Ethnicities. (2012) 13. 10.1177/1468796812463547

[63] Usami S. On the differences between general cross-lagged panel model and random-intercept cross-lagged panel model: interpretation of cross-lagged parameters and model choice. Struct Equat Model Multidiscipl J. (2021) 28:331–44. 10.1080/10705511.2020.1821690

[64] Lieberman C. Education and social change. Am Second Educ. (1977) 7:42–8.

[65] Paquet G,. Corporate culture governance: Canada in the Americas. (2006). Available online at: https://citeseerx.ist.psu.edu/viewdoc/download?doi=10.1.1.569.5997&rep=rep1&type=pdf

[66] Rogers D Berg I. Jr. Occupation and ideology: The case of the small businessman. Human Organ. (2008) 20:103–11. 10.17730/humo.20.3.5126514507322540

[67] Rudman DL. Situating occupation in social relations of power: Occupational possibilities, ageism and the retirement ‘choice'. South Afr J Occup Ther. (2015) 45:27–33. 10.17159/2310-3833/2015/v45no1a52015

[68] Read H. When and why to empathize with political opponents. Philosoph Stud. (2022). 10.1007/s11098-022-01837-y

[69] Jacobsmeier ML. From black and white to left and right: Race, perception of candidates' ideologies, and voting behavior in U.S. house elections. Polit Behav. (2015) 37:595–621. 10.1007/s11109-014-9283-3

[70] Okello WK. Organized anxiety: Respectability politics, John Henryism, and the paradox of Black achievement. Race Ethnic Educ. (2021) 2:523–41. 10.1080/13613324.2020.1798916

[71] Pitcan M Marwick AE Danah B. Performing a vanilla self: Respectability politics, social class, and the digital world. J Comp Mediat Commun. (2018) 23:163–79. 10.1093/jcmc/zmy008

[72] Rymhs D. ‘Docile bodies shuffling in unison': The prisoner as worker in canadian prison writing. Life Writ. (2009) 6:313–27. 10.1080/14484520903082967

[73] Benn-John J. (Re)colonizing black canadian women & routes to resistance. Race Gender Class. (2016) 23:150–65. Available online at: https://www.jstor.org/stable/26529195

[74] Glenn EN. Settler colonialism as structure: A framework for comparative studies of U.S. race and gender formation. Sociol Race Ethnic. (2015) 1:52–72. 10.1177/2332649214560440

[75] Harris PJ. Gatekeeping and remaking: The politics of respectability in african american women's history and black feminism. J Women's Hist. (2003) 15:212–20. 10.1353/jowh.2003.0025

[76] Heilman B,. The Man Box: A Study on Being a Young Man in the US, UK, Mexico. (2017). Available online at: https://promundoglobal.org/resources/man-box-study-young-man-us-uk-mexico/

